# Maternal Dexamethasone Treatment Alters Tissue and Circulating Components of the Renin-Angiotensin System in the Pregnant Ewe and Fetus

**DOI:** 10.1210/en.2015-1197

**Published:** 2015-06-03

**Authors:** Alison J. Forhead, Juanita K. Jellyman, Miles J. De Blasio, Emma Johnson, Dino A. Giussani, Fiona Broughton Pipkin, Abigail L. Fowden

**Affiliations:** Department of Physiology, Development and Neuroscience (A.J.F., J.K.J., M.J.D.B., E.J., D.A.G., A.L.F.), University of Cambridge, Cambridge CB2 3EG, United Kingdom; Department of Biological and Medical Sciences (A.J.F.), Oxford Brookes University, Oxford OX3 0BP, United Kingdom; and Department of Obstetrics and Gynaecology (F.B.P.), University of Nottingham, Nottingham NG5 1PB, United Kingdom

## Abstract

Antenatal synthetic glucocorticoids promote fetal maturation in pregnant women at risk of preterm delivery and their mechanism of action may involve other endocrine systems. This study investigated the effect of maternal dexamethasone treatment, at clinically relevant doses, on components of the renin-angiotensin system (RAS) in the pregnant ewe and fetus. From 125 days of gestation (term, 145 ± 2 d), 10 ewes carrying single fetuses of mixed sex (3 female, 7 male) were injected twice im, at 10–11 pm, with dexamethasone (2 × 12 mg, n = 5) or saline (n = 5) at 24-hour intervals. At 10 hours after the second injection, maternal dexamethasone treatment increased angiotensin-converting enzyme (ACE) mRNA levels in the fetal lungs, kidneys, and heart and ACE concentration in the circulation and lungs, but not kidneys, of the fetuses. Fetal cardiac mRNA abundance of angiotensin II (AII) type 2 receptor decreased after maternal dexamethasone treatment. Between the two groups of fetuses, there were no significant differences in plasma angiotensinogen or renin concentrations; in transcript levels of renal renin, or AII type 1 or 2 receptors in the lungs and kidneys; or in pulmonary, renal or cardiac protein content of the AII receptors. In the pregnant ewes, dexamethasone administration increased pulmonary ACE and plasma angiotensinogen, and decreased plasma renin, concentrations. Some of the effects of dexamethasone treatment on the maternal and fetal RAS were associated with altered insulin and thyroid hormone activity. Changes in the local and circulating RAS induced by dexamethasone exposure in utero may contribute to the maturational and tissue-specific actions of antenatal glucocorticoid treatment.

In clinical practice, synthetic glucocorticoids, such as dexamethasone, are administered routinely to pregnant women at risk of preterm delivery in order to promote fetal maturation and neonatal survival (1, 2). These drugs mimic the normal rise in endogenous glucocorticoids seen in the fetus near term by promoting structural and functional changes in fetal tissues in preparation for life after birth (3). Over the last 40 years, antenatal glucocorticoid therapy has improved survival of the premature infant and has reduced markedly the incidence of many disorders associated with preterm delivery, such as respiratory distress syndrome (2, 4). The clear beneficial effects of maternal glucocorticoid treatment have been offset, however, by evidence showing adverse consequences for growth and long-term blood pressure (BP) control, especially in infants exposed to multiple doses in utero (5–7). It is therefore important to understand the mechanisms of glucocorticoid action in the control of fetal growth and maturation.

Some of the effects of the glucocorticoids on the development of fetal tissues are mediated, in part, by other endocrine systems (8, 9). For example, in fetal sheep, endogenous and synthetic glucocorticoids stimulate the production of the active thyroid hormone, T_3_, and in turn, T_3_ promotes hepatic glycogen deposition and gluconeogenic enzyme activity in preparation for blood glucose control at birth (10–13). The renin-angiotensin system (RAS) is functional in the fetus from relatively early in gestation (14, 15) and is known to have an important role in the growth and development of specific tissues, as well as in the regulation of renal and cardiovascular function in utero (16–18). A number of maturational changes are observed in the fetal RAS near term, some of which are regulated by the prepartum surge in endogenous glucocorticoids (19–22). In addition, direct administration of dexamethasone to the sheep fetus increases both pulmonary and circulating concentrations of angiotensin-converting enzyme (ACE) in association with a rise in fetal arterial BP (23). However, the effect of maternal dexamethasone treatment, in a regime similar to that used in clinical practice, on the components of the RAS in the pregnant mother and fetus during late gestation is unknown in any species.

Therefore, the aim of this study was to investigate the acute effect of maternal dexamethasone treatment, in clinically relevant doses, on various components of the RAS in the pregnant ewe and fetus. The study hypothesized that synthetic glucocorticoid administration to the pregnant ewe would stimulate components of both the maternal and fetal RAS with potential consequences for fetal development.

## Materials and Methods

### Animals

Ten Welsh Mountain ewes carrying singleton fetuses of known gestational age were used in this study. There were 3 female and 7 male fetuses. The ewes were maintained on 200 g kg^−1^ concentrates daily (sheep nuts number 6; 18% protein and 10 MJ/kg; H & C Beart Ltd) with free access to hay, water and a salt-lick block. All experimental procedures were carried out in accordance with the United Kingdom Animals (Scientific Procedures) Act 1986 and approved by the research ethics committee at the University of Cambridge.

### Experimental procedures

From 125 days of gestation, all of the ewes were injected twice im with either dexamethasone (2 × 12 mg in 2-mL 0.9% NaCl, n = 5) or saline (2-mL 0.9% NaCl, n = 5) at 24-hour intervals. The experimental regime of dexamethasone treatment was similar to that recommended in human clinical practice by the Royal College of Obstetricians and Gynaecologists (24). At 10 hours after the second injection, the fetuses were delivered by caesarean section under general anesthesia (20 mg kg^−1^ sodium pentobarbitone iv). This time point was chosen so that data were obtained when the fetal dexamethasone concentration was comparable with previous studies that examined the cardiovascular effects of 1) maternal dexamethasone treatment and 2) direct fetal dexamethasone infusion in chronically catheterized fetuses (25, 26). The plasma dexamethasone concentration in the sheep fetus at this time point was approximately one-fifth of that measured in umbilical arterial blood samples taken from human infants at caesarean section after maternal dexamethasone treatment (27). Before anesthesia at between 9 and 10 am, 10-mL blood samples were obtained from the ewes by jugular venepuncture. At delivery, 10-mL blood samples were taken by venepuncture of the umbilical artery, and a number of tissues were collected from the ewes and fetuses after the administration of a lethal dose of barbiturate (200 mg kg^−1^ sodium pentobarbitone iv). Samples of lung, kidney, and heart from the fetus, and lung and kidney, but not heart, from the ewe, were immediately frozen in liquid nitrogen and stored at −80°C until analysis.

### Biochemical analyses

#### Plasma hormone concentrations

All blood samples were immediately placed into EDTA-containing tubes and centrifuged for 5 minutes at 1000*g* and 4°C. The plasma aliquots were stored at −20°C until analysis. Plasma angiotensinogen and renin concentrations were measured by RIA as described previously (28, 29). The lower limits of detection were 0.01 μg mL^−1^ for angiotensinogen and 0.5 pg mL^−1^ h^−1^ for renin. Plasma concentrations of cortisol, T_4_, T_3_, and insulin were measured by RIA or ELISA as detailed and published in these animals previously (12, 30).

#### RAS protein concentrations

Tissue and plasma ACE concentrations (as a proxy measure of activity) were determined by a spectrophotometric enzyme assay as described previously (20, 22). Tissue ACE concentration was expressed as nanomoles of hippurate generated per min per mg protein, whereas plasma ACE concentration was measured in U l^−1^ where 1 U equals 1 μmol of hippurate generated in 1 minute. Protein levels of the angiotensin (AII) type 1 receptor (AT_1_R) and AT_2_R subtypes were determined in maternal lung and renal cortex, and fetal lung, heart, and renal cortex, by Western blotting as detailed previously (31). The primary antibodies used were both rabbit polyclonal antibodies to epitopes on the human AT_1_R (0.2-μg/mL 306, sc-579; Santa Cruz Biotechnology, Inc) and the human AT_2_R (0.04-μg/mL H-143, sc-9040; Santa Cruz Biotechnology, Inc) (Supplemental Table 1). Membranes were analyzed with Ponceau S to normalize for protein loading as validated previously (32). Proteins were quantified using ImageJ software (National Institutes of Health; http://rsb.info.nih.gov/ij/), and ratios of protein content were arcsine transformed before statistical analysis.

#### RAS mRNA abundance

Tissue mRNA abundance of renin, ACE, AT_1_R, and AT_2_R were measured by TaqMan quantitative RT-PCR. Frozen samples of tissue (15 mg) were placed in Lysing Matrix-D tubes (MP Biomedicals) with 170-μL lysis/binding solution from a MagMax96 Total RNA Isolation kit (Life Technologies) and 0.75-μL β-mercaptoethanol, and homogenized using a FastPrep-24 (MP Biomedicals). After homogenisation, 106-μL 100% isopropanol was added to each sample. Samples were placed into a MagMAX96 system (Applied Biosystems) where RNA was isolated and deoxyribonuclease (DNase)-treated (TURBO DNase) using the MagMAX96 Total RNA Isolation kit (Life Technologies). Sample RNA yields and purities were assessed by a Nanodrop (Thermo Fisher). Ratios of absorption (260/280nm) of all preparations were between 1.8 and 2.0.

Reverse transcription of mRNA was carried out using a PCR Express machine (Thermo Fisher) and materials from Promega and Invitrogen. For each sample, 5 μL of DNase-treated RNA was mixed with 1-μL random primers, 1-μL deoxyribonucleotide triphosphate mix, and 5-μL ribonuclease-free water, and incubated at 65°C for 2 minutes. A master reverse transcription mix was made, consisting of 4-μL first strand buffer, 2-μL dithiothreitol, 1-μL RNaseOUT, and 1-μL Superscript II enzyme. The samples were incubated at room temperature for 5 minutes, at 42°C for 50 minutes and at 70°C for 15 minutes.

TaqMan quantitative real-time polymerase chain reaction (qRT-PCR) was performed to measure mRNA abundance of target genes in tissue samples. Samples were analyzed using a TaqMan 7900HT and data were acquired and processed with Sequence Detector version 2.3 software (Applied Biosystems). TaqMan Master Mix (5 μL), 0.5-μL target gene probe and primer set, and 3.5-μL water, were added to each well of a 96-well high-throughput plate (Applied Biosystems). In addition, 1-μL tissue cDNA at 1:20 dilution was added to each well apart from the nontemplate controls, where 1 μL of water was added. The sequences of the TaqMan qRT-PCR probes for renin, ACE, AT_1_R, and AT_2_R are listed in Table 1. Each tissue sample was measured in triplicate and normalized to the geometric mean of 2 housekeeping genes, glyceraldehyde 3-phosphate dehydrogenase, and cyclophilin A (Table 1). The mRNA levels of these housekeeping genes were not affected by maternal dexamethasone treatment. For each assay, a negative control without cDNA was included to ensure that amplicon contamination had not occurred in the reaction. Cycle thresholds determined by qRT-PCR were analyzed by the delta-delta-cycle threshold method as all standard curves were linear and parallel.

**Table 1.** Primer and Reporter Sequences Used for TaqMan qRT-PCR in the Sheep

Gene	Forward Primer Sequence	Reverse Primer Sequence	Reporter Sequence	Reporter Dye
Renin	GGATCTGGGAAGGTCAAAGGTTTC	CGCCAAAGGTCTGTGTGACT	CCGCCCACAGTCACC	FAM
ACE	CCTTCCCGCTACAACTATGACT	GGACAACCGGAGGACAGATC	ATACTTGGTTCGAAGATACC	FAM
AT_1_R (bovine)	TaqMan gene expression assays (Assay ID Bt03213473_m1; part number 4331182; reporter sequence AGGTCTGCATCCAGGTGCATTTGGC)			FAM
AT_2_R	CTGTCATTTACCCCTTTCTGTCTCA	CAGACAAGCCATACACCAAACAAG	TTGCCAGGGATTTCT	FAM
Glyceraldehyde 3-phosphate dehydrogenase	GCTACACTGAGGACCAGGTT	AGCATCGAAGGTAGAAGAGTGAGT	CTCCTGCGACTTCAAC	FAM
Cyclophilin A	GGTTCCTGCTTTCACAGAATAATTCC	GTACCATTATGGCGTGTGAAGTCA	CACCCTGGCACATAAA	FAM

### Statistical analyses

A sample size of 5 animals was calculated in order to find a 2-fold difference in fetal pulmonary ACE concentration, assuming a SD of 0.26, and to achieve 99% power at the 5% significance level. This sample size calculation was based on mean and SD values measured in previous studies from this laboratory examining the effects of fetal dexamethasone treatment on pulmonary ACE concentration (23) (Sigmastat 3.5; Systat Software, Inc). All data are presented as mean ± SEM. The distributions of data for plasma and tissue measurements were assessed for normality by the Kolmogorov-Smirnov test, and compared between the treatment groups by Student's unpaired *t* test (parametric) or Mann-Whitney test (nonparametric), as appropriate. Relationships between the variables measured were determined by Pearson correlation and partial correlation analyses. The null hypothesis was rejected where *P* < .05.

## Results

### Fetus

**Figure 1.**
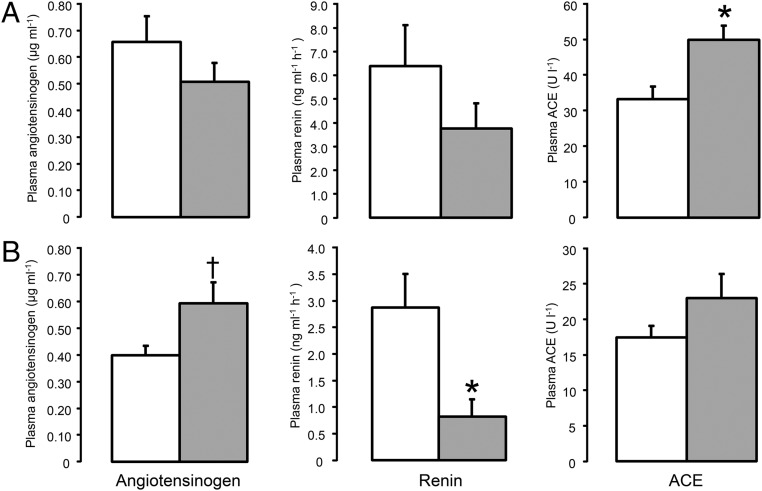
Plasma concentrations of angiotensinogen, renin, and ACE in the fetuses (A) and ewes (B) sampled at 10 hours after the second daily injection of either saline (□, n = 5) or dexamethasone (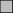, n = 5). Data are presented as mean values (±SEM). Significant difference from saline-treated group: *, *P* < .05; †, *P* = .06.

Within 10 hours of the second maternal injection of dexamethasone, the fetal plasma concentration of ACE increased significantly (*P* < .05) (Figure 1A). Plasma cortisol decreased, and T_3_ and insulin concentrations increased in the fetuses exposed to dexamethasone compared with those from the saline-treated ewes (*P* < .05) (Table 2). There were no significant differences in plasma concentrations of angiotensinogen, renin or T_4_ between the 2 groups of fetuses (Table 2 and Figure 1A).

**Table 2.** Plasma Concentrations of Cortisol, Thyroid Hormones (T_4_ and T_3_), and Insulin in the Ewes and Fetuses at 10 Hours After Saline or Dexamethasone Treatment

	Ewe		Fetus	
	Saline	Dexamethasone	Saline	Dexamethasone
Cortisol (ng mL^−1^)	57.8 ± 12.2	3.2 ± 0.1^a^	16.1 ± 2.8	10.0 ± 1.4^a^
T_3_ (ng mL^−1^)	1.13 ± 0.08	0.69 ± 0.06^a^	0.28 ± 0.06	0.70 ± 0.08^a^
T_4_ (ng mL^−1^)	52.1 ± 10.6	21.8 ± 5.3^a^	132.5 ± 11.7	115.0 ± 21.3
Insulin (ng mL^−1^)	0.19 ± 0.08	0.27 ± 0.05	0.25 ± 0.05	1.35 ± 0.31^a^

Data are presented as mean values (±SEM).

a Significantly different from saline-treated group, *P* < .05.

**Figure 2.**
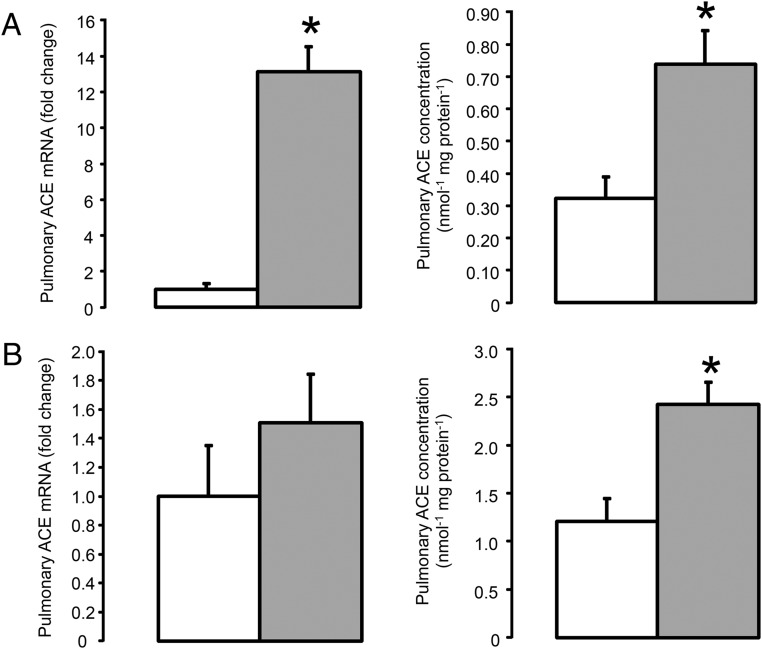
Pulmonary ACE mRNA and concentration in the fetus (A) and ewe (B) at 10 hours after saline (□, n = 5) or dexamethasone (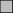, n = 5) treatment. Data are presented as mean values (±SEM); transcript data are presented as fold changes relative to the saline-treated group. Significant difference from saline-treated group: *, *P* < .05.

**Figure 3.**
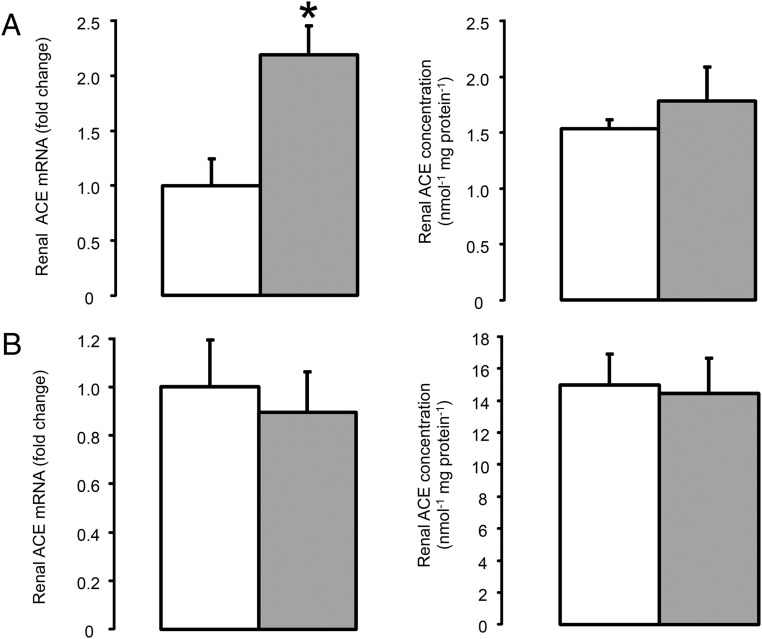
Renal ACE mRNA and concentration in the fetus (A) and ewe (B) at 10 hours after saline (□, n = 5) or dexamethasone (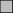, n = 5) treatment. Data are presented as mean values (±SEM); transcript data are presented as fold changes relative to the saline-treated group. Significant difference from saline-treated group: *, *P* < .05.

**Figure 4.**
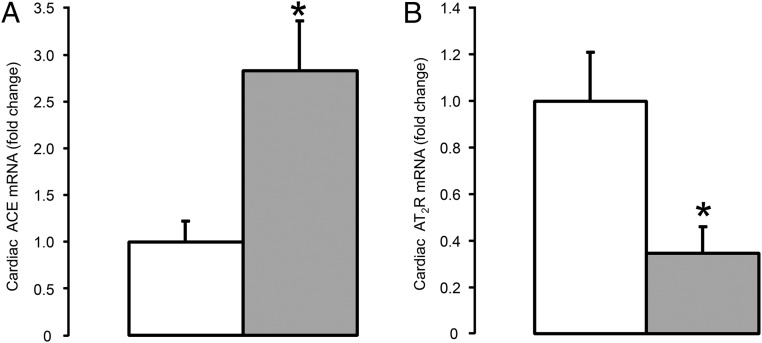
Cardiac ACE (A) and AT_2_R (B) mRNA levels in the fetus at 10 hours after saline (□, n = 5) or dexamethasone (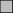, n = 5) treatment. Data are presented as mean fold changes (±SEM) relative to the saline-treated group. Significant difference from saline-treated group: *, *P* < .05.

Pulmonary ACE mRNA and ACE concentration were significantly greater in the fetuses exposed to dexamethasone compared with the control fetuses (*P* < .05) (Figure 2A). The mRNA levels of ACE in the fetal kidney and heart were also elevated by maternal dexamethasone treatment (*P* < .05) (Figures 3A and 4A), although renal ACE concentration was unchanged and cardiac ACE concentration was below the limit of assay detection in both groups of fetuses (0.10 nmol min^−1^ per mg protein^−1^) (Figure 3A).

Neither relative mRNA abundance nor protein content for AT_1_R and AT_2_R in the lungs and kidneys were significantly different between fetuses of mothers treated with dexamethasone or saline (Table 3). Renal renin mRNA abundance was also unchanged by maternal dexamethasone treatment (Table 3). In the fetal heart, no significant changes in AT_1_R mRNA level, or protein content of AT_1_R or AT_2_R, were seen after maternal dexamethasone treatment, although cardiac AT_2_R mRNA abundance was reduced in the fetuses exposed to dexamethasone (Table 3 and Figure 4B; *P* < .05).

**Table 3.** Relative Protein and mRNA Levels of Renin, AT_1_R, and AT_2_R in Fetal and Maternal Lung and Kidney at 10 Hours After Saline or Dexamethasone Treatment

	Ewe		Fetus	
	Saline	Dexamethasone	Saline	Dexamethasone
Lung mRNA				
AT_1_R	1.00 ± 0.29	1.69 ± 0.50	1.00 ± 0.25	1.57 ± 0.25
AT_2_R	1.00 ± 0.79	2.21 ± 1.15	1.00 ± 0.22	1.56 ± 0.43
Lung protein				
AT_1_R	1.00 ± 0.33	1.09 ± 0.26	1.00 ± 0.05	1.19 ± 0.09
AT_2_R	1.00 ± 0.08	1.03 ± 0.15	1.00 ± 0.15	1.15 ± 0.17
Kidney mRNA				
Renin	1.00 ± 0.24	0.53 ± 0.19	1.00 ± 0.33	1.37 ± 0.50
AT_1_R	1.00 ± 0.51	0.76 ± 0.29	1.00 ± 0.43	1.11 ± 0.49
AT_2_R	1.00 ± 0.59	0.21 ± 0.06	1.00 ± 0.40	0.57 ± 0.23
Kidney protein				
AT_1_R	1.00 ± 0.08	0.99 ± 0.07	1.00 ± 0.11	0.95 ± 0.10
AT_2_R	1.00 ± 0.08	1.16 ± 0.08	1.00 ± 0.10	1.21 ± 0.13
Heart mRNA				
AT_1_R	NA	NA	1.00 ± 0.28	1.20 ± 0.19
AT_2_R	NA	NA	1.00 ± 0.16	1.23 ± 0.09
Heart protein				
AT_1_R	NA	NA	1.00 ± 0.10	1.15 ± 0.07
AT_2_R	NA	NA	1.00 ± 0.21	0.34 ± 0.11^a^

Data are presented as mean fold changes (±SEM) relative to the saline-treated group. NA, not available.

^a^ Significant difference from saline-treated group, *P* < .05.

When observations from all of the fetuses were considered, a significant positive correlation was observed between pulmonary ACE concentration and circulating ACE levels (r = +0.77, *P* < .001, n = 10). Significant negative correlations were observed between plasma cortisol concentration and both pulmonary ACE mRNA abundance and plasma ACE concentration (Table 4). Plasma renin concentration was negatively correlated with plasma ACE concentration in the fetuses (Table 4). In addition, plasma T_3_ correlated with circulating and pulmonary ACE concentrations, and with ACE mRNA abundance in the fetal lungs, kidneys, and heart (Table 4). Significant positive relationships were also seen between plasma insulin concentration and various components of the fetal RAS (Table 4). Partial correlation analyses showed that pulmonary ACE concentration and renal ACE mRNA were positively associated with plasma T_3_ concentration (r = +0.67, *P* < .05, n = 10 and r = +0.72, *P* < .05, n = 10, respectively), independent of plasma insulin; all other partial correlations failed to identify a single significant independent factor when multiple hormones correlated with RAS components.

**Table 4.** Correlation Coefficients From Relationships Between Plasma Hormone Concentrations in the Fetuses, and ACE mRNA and Concentrations in the Fetal Circulation and Tissues

	Plasma ACE	Lung ACE mRNA	Lung ACE Concentration	Kidney ACE mRNA	Kidney ACE Concentration	Heart ACE mRNA
Cortisol	−0.71^a^	−0.67^a^	NS	NS	NS	NS
T_3_	+0.63^a^	+0.81^b^	+0.82^b^	+0.84^b^	NS	+0.88^b^
Insulin	+0.75^a^	+0.80^a^	+0.66^a^	+0.65^a^	+0.69^a^	NS
Renin	−0.72^a^	NS	NS	NS	NS	NS

NS, not significant. Pearson correlation, n = 10.

a *P* < .05.

b *P* < .005.

### Ewe

In the ewes treated with dexamethasone, plasma concentrations of cortisol, renin, T_3_, and T_4_ were suppressed, and plasma insulin increased, within 10 hours of the second injection (Table 2 and Figure 2B; *P* < .05). Plasma angiotensinogen concentration was increased by dexamethasone administration, but this just failed to reach statistical significance (*P* = .059) (Figure 1B). Maternal dexamethasone treatment had no significant effect on plasma ACE concentration (Figure 1B).

Pulmonary ACE concentration was increased in the dexamethasone-treated ewes compared with those treated with saline (*P* < .05) (Figure 2B). However, there was no significant effect of dexamethasone administration on ACE mRNA abundance in the maternal lungs (Figure 2B). Renal ACE mRNA and ACE concentrations were also unchanged by maternal dexamethasone treatment (Figure 3B). Maternal dexamethasone treatment had no significant effect on the gene transcript or protein levels of the AII receptors in the lungs and kidneys, or renin mRNA abundance in the kidneys of the pregnant ewes (Table 3).

Using data from all ewes, significant inverse correlations were observed between pulmonary ACE concentration and plasma concentrations of both cortisol (r = −0.89, *P* < .001, n = 10) and T_4_ (r = −0.80, *P* < .005, n = 10). Partial correlation analyses showed that pulmonary ACE concentration was inversely associated with plasma T_4_ concentration (r = −0.74, *P* < .05, n = 10), independent of plasma cortisol concentration. Plasma renin concentration was correlated with renal renin mRNA abundance (r = +0.70, *P* < .05, n = 10) in the pregnant ewes. Significant positive relationships were also seen between plasma T_3_ concentration and both renal renin mRNA abundance (r = +0.67, *P* < .05, n = 10) and plasma renin concentration (r = +0.75, *P* < .01, n = 10).

## Discussion

### Effects of maternal dexamethasone treatment on the RAS in utero

The present study demonstrates for the first time that maternal dexamethasone treatment, at a dose equivalent to that used in clinical practice, alters various components of the RAS in both the pregnant ewe and fetus. Administration of the synthetic glucocorticoid up-regulated ACE mRNA abundance in a variety of fetal tissues; it also increased pulmonary ACE concentration in both the pregnant ewe and fetus and ACE concentration in the fetal circulation. In the pregnant ewe, plasma angiotensinogen tended to increase in response to dexamethasone administration, and plasma renin was suppressed, which suggested negative feedback control by activation of the AT_1_R in the kidney.

In the present study, the increments in circulating and pulmonary ACE concentrations observed in the fetuses after maternal dexamethasone treatment were similar to those seen previously in sheep fetuses infused directly with the synthetic glucocorticoid (23). In addition, both fetal and maternal routes of dexamethasone administration had no significant effect on renal ACE concentration in the sheep fetus (23). The present findings indicate that maternal dexamethasone treatment elevated pulmonary and circulating ACE concentration in utero, at least in part, by increasing ACE mRNA abundance in the lungs and other fetal tissues. At 10 hours after the second injection of dexamethasone to the pregnant ewe, ACE mRNA levels in the fetal kidney and heart had increased without any change in enzyme concentration. The duration of exposure to the synthetic glucocorticoid, and/or the timing of tissue collection, however, may have been too short to observe significant effects on protein translation in these fetal organs.

The changes in plasma and pulmonary ACE concentration, and cardiac ACE mRNA, induced in utero by maternal dexamethasone treatment were similar to the normal maturational changes seen in sheep fetuses close to term (22, 33). In the lungs, ACE is localized to the vascular endothelium and for most of gestation, both pulmonary blood flow and ACE concentration in the fetal lungs are relatively low. However, pulmonary ACE concentration in the fetus increases near term and this appears to be driven by the prepartum glucocorticoid surge as part of the preparation for extrauterine life (22). Neonatal plasma AII concentrations after vaginal delivery are much higher than after caesarean section in human infants (34), suggesting that the capacity to convert AI to AII is activated by exposure to endogenous glucocorticoids before birth in preparation for pulmonary vasodilatation and increased pulmonary blood flow and delivery of AI after birth.

Developmental changes in AII receptor expression are also seen in the fetus towards term. The relative expression of AII receptor subtypes in a variety of fetal tissues shifts with gestational age from widespread AT_2_R abundance to tissue-specific and predominant localization of AT_1_R (35). In the heart of the sheep fetus, mRNA abundance and receptor density of the AT_2_R receptor are high from at least midgestation and decrease over the perinatal period (36, 37). The reduction in cardiac AT_2_R mRNA level induced by dexamethasone in the present study indicates that the normal developmental decline in AT_2_R expression seen in the fetal heart near term may be a glucocorticoid-dependent event.

### Mechanisms of glucocorticoid action on the RAS

The mechanisms of glucocorticoid action on the RAS in the pregnant ewe and fetus may be direct and/or indirect involving coincident changes in other endocrine systems, such as insulin and the thyroid hormones. The effects of antenatal glucocorticoid treatment on the RAS observed in the present study are unlikely to be the consequence of fetal hypoxaemia or hypotension. In the chronically catheterized pregnant ewe and fetus, the same protocol of maternal dexamethasone treatment does not influence fetal blood gas status and causes a small, but significant, rise in arterial BP (26, 38).

Dexamethasone may have direct effects on the genes for angiotensinogen, ACE and the AT_2_R. A glucocorticoid-response element in the angiotensinogen gene is an important regulator of angiotensinogen synthesis (39); thus, the near-significant rise in maternal plasma angiotensinogen may have been directly stimulated by dexamethasone treatment. Glucocorticoid-response elements have also been identified close to a promoter region in the murine and human ACE gene (40), and dexamethasone has been shown previously to promote ACE mRNA abundance and enzyme activity in rabbit alveolar macrophages, bovine pulmonary artery endothelial cells and rat cardiac fibroblasts studied in vitro (41–43). Moreover, dexamethasone increases ACE mRNA abundance and enzyme activity in cultured rat aortic smooth muscle cells by stabilization of mRNA as well as enhanced gene transcription (44). In rats, multiple glucocorticoid-response elements are localized near to the regulatory region of the AT_2_R gene, which have inhibitory influences on promoter activity and AT_2_R gene expression (45). Indeed, AT_2_R mRNA and protein levels in hearts isolated from fetal rats are suppressed by 48 hours of dexamethasone treatment in vitro (45).

The present study was part of a larger project examining the effects of maternal dexamethasone treatment on fetal growth and development and in which plasma concentrations of insulin and the thyroid hormones were measured (12, 30). Significant associations were observed between circulating concentrations of T_3_ in the fetus and plasma and pulmonary ACE concentrations, and the transcript levels of ACE in the fetal lungs, kidneys, and heart. These findings support the suggestion that T_3_ may have an important role in mediating the regulatory effects of glucocorticoids on tissue and circulating ACE expression in utero (46). Indeed, in fetal sheep, experimental thyroid hormone deficiency prevents the normal developmental rise in pulmonary and renal ACE concentration near term, and exogenous T_3_ infusion has been shown to increase ACE concentration in the fetal lungs, but not kidneys (46). Previous studies, however, have shown that maternal dexamethasone treatment has differential effects on the thyroid hormone axis in the pregnant ewe and fetus (12). In the sheep fetus, synthetic and endogenous glucocorticoids activate the production of T_3_, whereas in the mother, the thyroid hormone axis is suppressed (11, 12). Therefore, the rise in pulmonary ACE concentration seen in the pregnant ewe treated with dexamethasone may be the direct consequence of glucocorticoid, rather than T_3_, action. Alternatively, dexamethasone and/or T_3_ may have different and tissue-specific effects on ACE expression in the fetus and mother. In adult rats, dexamethasone treatment causes a rise in ACE concentration in the lungs, but not renal cortex or medulla, and T_3_ administration reduces pulmonary ACE, while increasing renal and circulating ACE, concentrations (47).

### Implications of altered RAS activity induced by maternal dexamethasone treatment

Changes in the activity of the RAS in utero after maternal dexamethasone treatment may contribute to the maturation of fetal tissues induced clinically by synthetic glucocorticoids. In addition, alterations in fetal RAS activity may have both local and endocrine effects on growth and maturation before birth (48). Although a limitation of this study was that circulating and tissue concentrations of AII, and downstream signaling pathways, were not determined, the increase in pulmonary ACE concentration is likely to result in enhanced production of AII locally in the lungs of the ewe and fetus. Local production of AII in the fetal lungs has been shown to promote maturation of pulmonary structure, including vascularization and airway branching (49, 50), and may mediate, in part, some of the beneficial effects of antenatal synthetic glucocorticoids on the developing lungs.

Activation of ACE mRNA, and suppression of AT_2_R mRNA, abundances in the fetal heart by dexamethasone may influence the development of cardiac structure and function in utero, if the mRNA levels were to translate to altered protein expression in the longer term. The developmental processes of growth and differentiation in fetal cardiomyocytes are sensitive to glucocorticoids and thyroid hormones (51), and the mechanisms of hormone action may involve changes in local AII activity (52, 53). It is increasingly recognized that, whereas antenatal synthetic glucocorticoid treatment can be life-saving for the infant when delivery occurs preterm, there may also be adverse sequelae reaching into adulthood (54). Indeed, dexamethasone-induced changes in the fetal RAS, especially within the heart and kidney, may have consequences for the regulation of arterial BP in both fetal and postnatal life. In fetal sheep, exposure to dexamethasone either by direct fetal infusion or by maternal treatment causes an increase in arterial BP and modifies the cardiovascular responses to hypoxaemia induced experimentally in utero (23, 26, 38). Previously, arterial BP was found to correlate with pulmonary ACE concentration in sheep fetuses infused with either dexamethasone or saline (23). Pulmonary ACE is responsible both for the production of vasoconstrictive AII and for the degradation of the vasodilator bradykinin, and the RAS is known to have an important role in the control of fetal BP by peripheral and central mechanisms (55–57). Furthermore, the RAS has been implicated in the developmental programming of hypertension in sheep and rodent offspring exposed to glucocorticoids in utero (58–60). In conclusion, antenatal dexamethasone treatment stimulates components of the maternal and fetal RAS, and suppresses fetal cardiac AT_2_R mRNA levels, in the sheep. These changes may influence maturation of the developing lungs, heart, and kidney and may have acute and long-term consequences for the regulation of arterial BP.
